# Clinical Data Miner: An Electronic Case Report Form System With Integrated Data Preprocessing and Machine-Learning Libraries Supporting Clinical Diagnostic Model Research

**DOI:** 10.2196/medinform.3251

**Published:** 2014-10-20

**Authors:** Arnaud JF Installé, Thierry Van den Bosch, Bart De Moor, Dirk Timmerman

**Affiliations:** ^1^Department of Electrical Engineering ESATSTADIUS Center for Dynamical Systems, Signal Processing and Data AnalyticsKU LeuvenLeuvenBelgium; ^2^iMinds Medical ITLeuvenBelgium; ^3^Department of Obstetrics and GynecologyUZ LeuvenKU LeuvenLeuvenBelgium; ^4^Department of Development and RegenerationUZ LeuvenKU LeuvenLeuvenBelgium

**Keywords:** data collection, machine-learning, clinical decision support systems, data analysis

## Abstract

**Background:**

Using machine-learning techniques, clinical diagnostic model research extracts diagnostic models from patient data. Traditionally, patient data are often collected using electronic Case Report Form (eCRF) systems, while mathematical software is used for analyzing these data using machine-learning techniques. Due to the lack of integration between eCRF systems and mathematical software, extracting diagnostic models is a complex, error-prone process. Moreover, due to the complexity of this process, it is usually only performed once, after a predetermined number of data points have been collected, without insight into the predictive performance of the resulting models.

**Objective:**

The objective of the study of Clinical Data Miner (CDM) software framework is to offer an eCRF system with integrated data preprocessing and machine-learning libraries, improving efficiency of the clinical diagnostic model research workflow, and to enable optimization of patient inclusion numbers through study performance monitoring.

**Methods:**

The CDM software framework was developed using a test-driven development (TDD) approach, to ensure high software quality. Architecturally, CDM’s design is split over a number of modules, to ensure future extendability.

**Results:**

The TDD approach has enabled us to deliver high software quality. CDM’s eCRF Web interface is in active use by the studies of the International Endometrial Tumor Analysis consortium, with over 4000 enrolled patients, and more studies planned. Additionally, a derived user interface has been used in six separate interrater agreement studies. CDM's integrated data preprocessing and machine-learning libraries simplify some otherwise manual and error-prone steps in the clinical diagnostic model research workflow. Furthermore, CDM's libraries provide study coordinators with a method to monitor a study's predictive performance as patient inclusions increase.

**Conclusions:**

To our knowledge, CDM is the only eCRF system integrating data preprocessing and machine-learning libraries. This integration improves the efficiency of the clinical diagnostic model research workflow. Moreover, by simplifying the generation of learning curves, CDM enables study coordinators to assess more accurately when data collection can be terminated, resulting in better models or lower patient recruitment costs.

## Introduction

### Saving Lives With Early Detection

**Figure 1:**
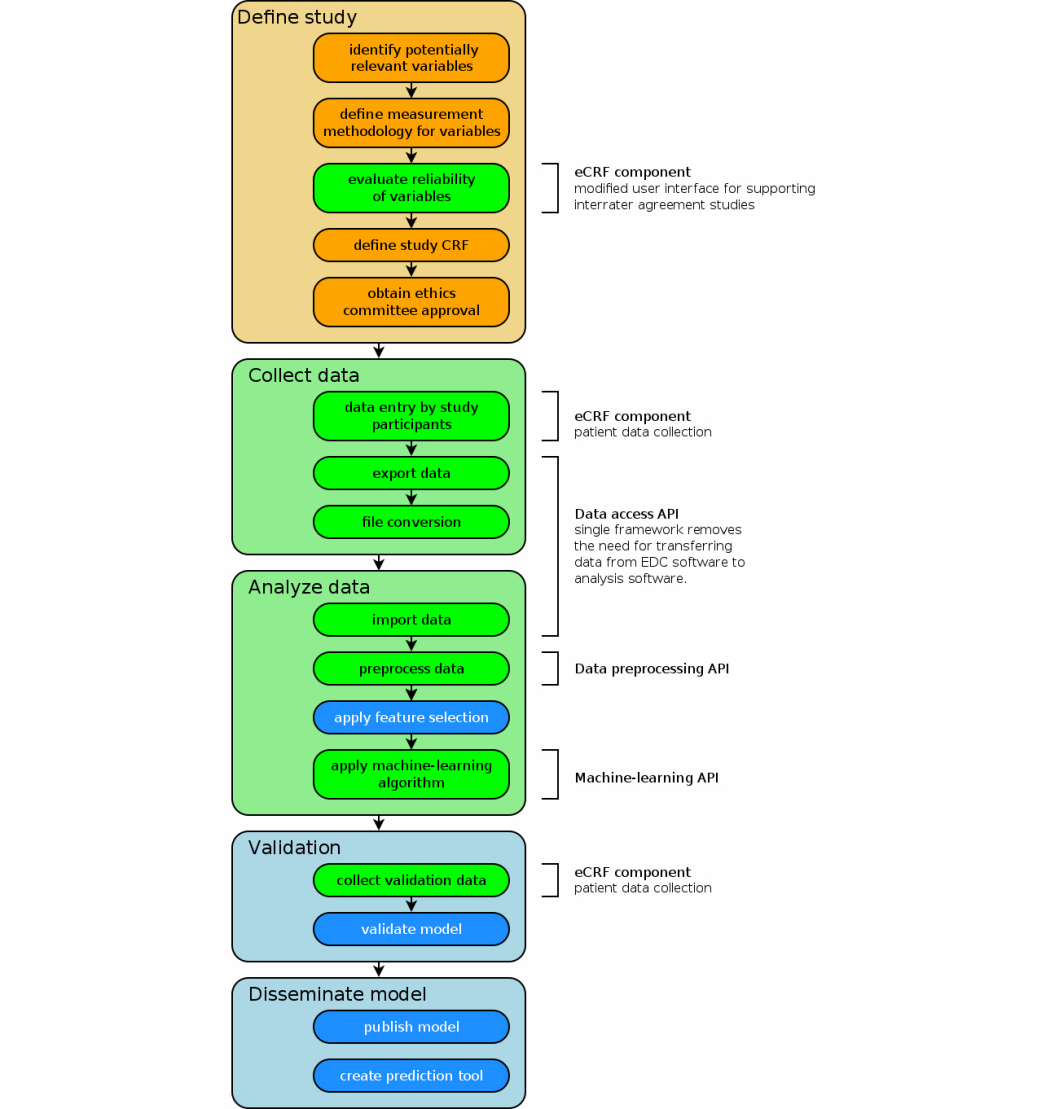
Typical workflow of clinical diagnostic model research. The Clinical Data Miner software framework improves support for the steps indicated in green. Support for steps marked in blue is planned for future work. (Abbreviations used: CRF=case report form; eCRF=electronic CRF; API=application programming interface.).

Many diseases, including cancer, may be cured or managed, if diagnosed sufficiently early. However, a lot of these go undetected, resulting in many avoidable deaths. A report from 2009 estimates that, for the case of cancer in the United Kingdom alone, five to ten thousand deaths could be prevented yearly through early diagnosis [1]. Improving early diagnosis could thus beneficially affect patient outcomes, but is impeded by several factors, including cost and invasiveness of relevant diagnostic procedures. Thus, one of the aims of clinical diagnostic model research is to find diagnostic models with good predictive performance, using the cheapest and least invasive means possible. Examples of such research are the studies organized by the International Ovarian Tumor Analysis [2-5] and International Endometrial Tumor Analysis (IETA) [6] consortia, which investigate diagnostic models for ovarian and endometrial tumors, respectively. Figure 1 shows a typical clinical diagnostic model research workflow.

### Software to Support Clinical Diagnostic Model Research Workflow

Several software packages exist to support the clinical diagnostic model research workflow. Electronic case report form (eCRF) systems, such as REDCap [7] or the open-source OpenClinica, enable the collection of patient data. Compared with paper-based data collection, such systems reduce data error rates [8], and, according to a costs simulation study, enable cost reductions between 49% and 62% [9]. As a result, their use has greatly increased over the past decades, with reports of 41% out of 259 Canadian trials using electronic data capture software [10]⁠, and of 79.6% (417/524) of Hong Kong private physicians using electronic medical records [11]⁠.

Meanwhile, mathematical packages such as R [12], Matlab [13], or WEKA [14,15] support data analysis. Their inclusion of machine-learning techniques enables the extraction of sophisticated diagnostic models from patient data, with high predictive performance.

However, several steps in the clinical diagnostic model research workflow introduce unnecessary complexity. Data have to be extracted from the eCRF system, and imported back into data analysis software. These steps may lead to conversion issues, requiring manual inspection of the result. Furthermore, any case report form (CRF) structure information is lost in the process. For data preprocessing transformations, such as the replacement of categorical variables with dummy variables [16]⁠, the lack of CRF structure information requires either manual selection or the use of heuristics for determining which variables need to be transformed, both of which are prone to errors. Other transformations, such as dealing with structurally missing variables, can only be performed manually.

Moreover, the complexity of the data analysis step discourages intermediate assessments of predictive performance. As a result, clinical diagnostic model research usually relies on Monte Carlo simulations [17] or rules of thumb [18] for sample size requirements estimates. These may be both over and underestimated, leading to patient recruitment that is more expensive than needed, or to models with insufficient predictive performance, respectively.

We implemented the Clinical Data Miner (CDM) software framework [19]⁠ to support the studies organized by the IETA consortium [6]⁠. In doing so, we aimed to create a generic, multi-centric platform that avoids the aforementioned inefficiencies, with a user interface that can be integrated in various computing environments, such as mobile phones or hospital information systems (HIS).

## Methods

### Component Overview

In order to improve support for clinical diagnostic model research in general, and the IETA studies in particular, the CDM software framework consists of an eCRF component and a data analysis component. This section introduces the eCRF and data analysis components in more detail, discusses the methodology used in their development, and explains the modalities of a survey we conducted to examine user satisfaction with CDM's eCRF component.

### Electronic Case Report Form Component

**Figure 2:**
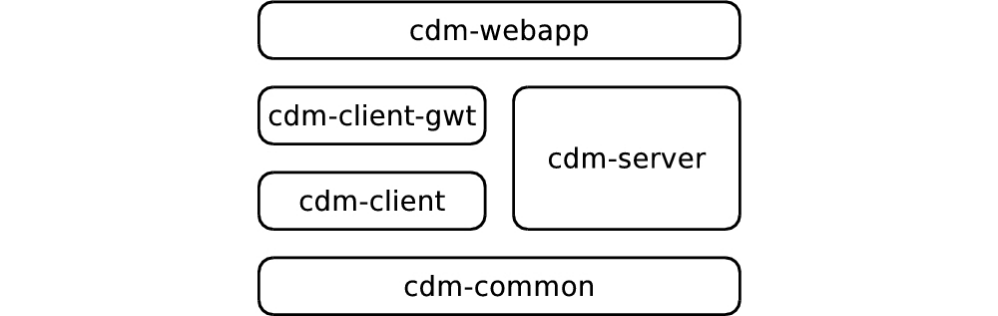
In Clinical Data Miner (CDM)'s layered architecture, module cdm-common contains functionality common to client and server. The server code is implemented in module cdm-server, while client code is further split into user interface logic (cdm-client) and user interface presentation (cdm-client-gwt). Finally, cdm-webapp combines the modules and provides CDM's entry point.

CDM's eCRF component parses CRFs from external files, using a spreadsheet format similar to that of OpenClinica. Defining CRFs by parsing external files enables support for generic studies. In order to simplify the organization of multi-center studies, CDM's eCRF component exhibits a client-server architecture, with a Web-based user interface at the client side. This client-server architecture is reflected in the eCRF component's modular design. Figure 2 shows this, with separate modules for client and server code. The design further separates user interface *logic* (cdm-client) and user interface *presentation* (cdm-client-gwt). The latter separation offers the possibility to implement alternative interfaces, such as a mobile phone app, or a user interface integrated in a HIS.

### Data Analysis Component

**Figure 3:**
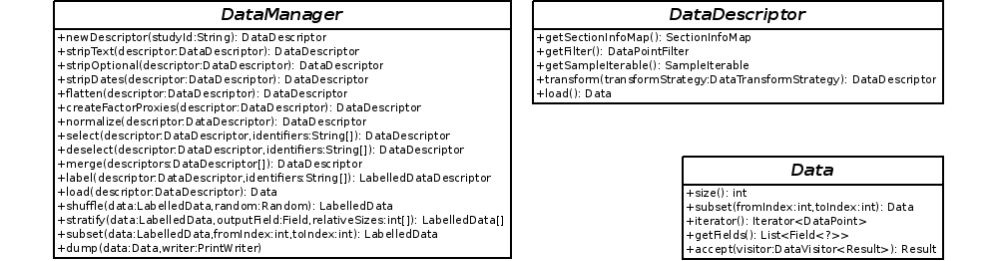
The DataManager application programming interfaces includes methods to access and preprocess data.

**Figure 4:**
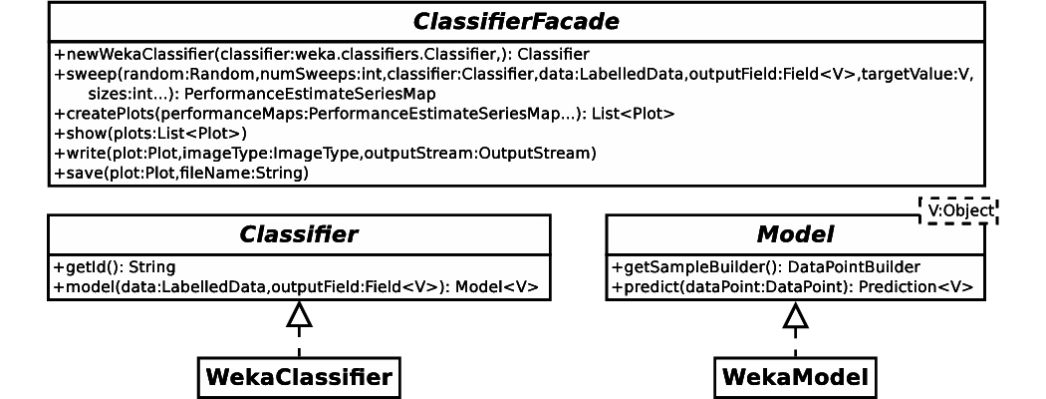
Unified Modeling Language diagram of Clinical Data Miner (CDM)'s machine-learning application programming interfaces. ClassifierFacade is the entry point to CDM's machine-learning functionality, which operates on Classifier objects to obtain Model objects.

CDM includes capabilities for analyzing data, consisting of Java libraries for data querying and preprocessing, and the application of supervised machine-learning techniques. The simplified Unified Modeling Language diagrams from Figures 3 and 4 illustrate the application programming interfaces (APIs) of these libraries. Here, the DataManager class from Figure 3 represents CDM's entry point to its data querying and preprocessing capabilities, while ClassifierFacade in Figure 4 provides access to its machine-learning capabilities.

The integration of an eCRF component with these data analysis libraries in a single system allows one to avoid exporting data from an eCRF system to import them back into data analysis software, eliminating potential conversion issues.

This integration additionally provides CDM's data preprocessing methods with direct access to CRF structure information. Instead of relying on manual input or heuristics, this direct access to CRF structure information enables preprocessing data with exact knowledge of type and dependency information for all variables. The createFactorProxies() preprocessor, for example, uses type knowledge of a CRF's variables to transform all categorical variables into sets of dummy variables [16]. Preprocessors such as flatten(), on the other hand, use information about dependencies between variables to convert data points with structurally missing variables to vectors. These are variables that may be missing depending on the value of a parent variable, as is the case for the variable “years past menopause” for patients with variable “menopausal status” set to “premenopausal”. By converting data points with structurally missing variables to vectors, the flatten() method enables the use of a wider variety of classification algorithms, such as logistic regression [20] or Least-Squares Support Vector Machines [21,22], without the need for defining specialized kernel methods.

Using the newWekaClassifier() method, the ClassifierFacade interface from Figure 4 constructs Classifier objects that provide access to the wealth of machine-learning algorithms and techniques available in the Weka toolbox [15]. Leveraging the Classifier interface, ClassifierFacade's sweep() method further enables the generation of learning curves, plotting the evolution of predictive performance measures, such as accuracy, sensitivity, specificity, or Area under the Receiver Operating Characteristic Curve, with respect to sample size.

Finally, CDM's Java libraries for data querying, data preprocessing, and machine-learning can be used interactively from within a Jython console by means of a set of Jython modules included in CDM.

### Software Development Methodology

We developed CDM using the Java programming language, leveraging the Google Window Toolkit (GWT) to translate client-side Java code to ECMAScript. In order to ensure good software quality, we developed CDM using a test-driven development (TDD) [22]⁠ process. We have integrated Cobertura [23]⁠ in CDM’s automated build process for test coverage monitoring. The resulting unit test suite allows automation of most of the quality assurance process required prior to the deployment of new releases.

Sound design and loose coupling are obtained through extensive use of design patterns [23] and dependency injection. The latter is achieved by means of the Spring framework server-side, and the Gin and Guice frameworks client-side.

### User Survey

In order to assess user satisfaction, we sent a survey to active CDM users, which included users who submitted at least ten patient entries through CDM's eCRF component, or who participated in an interrater agreement study organized using CDM's user interface, adapted for such studies. In total, we asked 42 clinicians to participate in the survey. The survey consisted of several questions examining user-friendliness, satisfaction with certain user interface elements, and software reliability.

## Results

### Electronic Case Report Form Component

**Figure 5:**
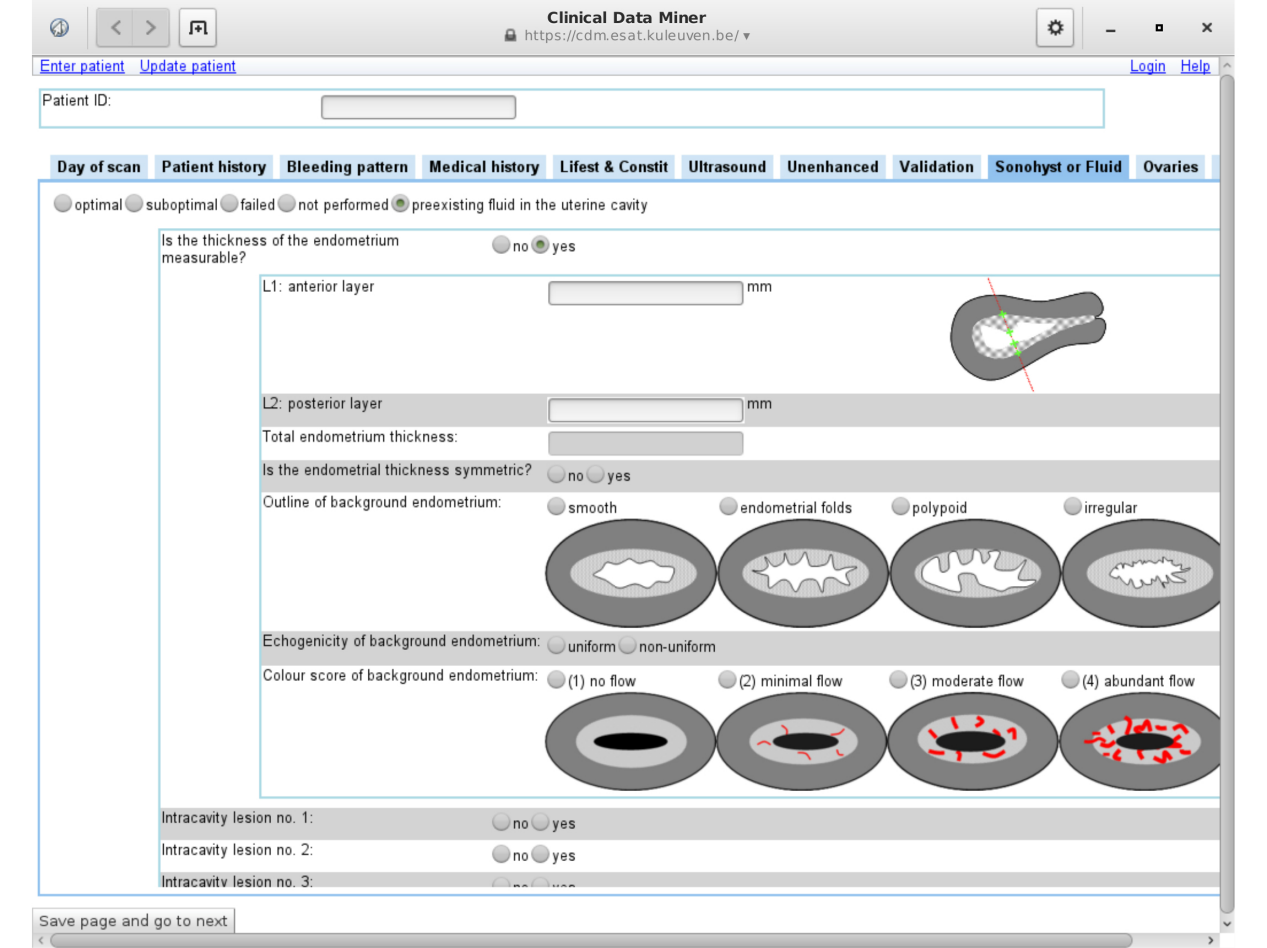
Clinical Data Miner (CDM)'s data collection user interface. The possibility to include pictograms in case report forms is particularly interesting for variables obtained from imaging modalities.

CDM has a client-server architecture. As Figure 5 illustrates, its current user interface is Web-based. This has enabled multi-center data collection in the context of the IETA studies. As Table 1 shows, CDM has collected 4035 patient entries so far for these studies, supplied by 39 participants from 24 different centers between May 2011 and September 2014.

**Table 1:** Number of patient entries collected by CDM for the IETA studies, between May 2011 and September 2014.

IETA	Complete entries	Total entries
#1	1600	2069
#3	641	787
#4	891	1179
Total	3132	4035

### Clinical Data Miner Architecture

CDM's modular, layered architecture enables parallel development of user interfaces for multiple computing environments, which in the future could thus include mobile phones or HIS. Moreover, the modularity of this architecture has facilitated the organization of interrater agreement studies that evaluate imaging modalities with the creation of a modified user interface that displays each imaging modality next to the questionnaire to be completed.

Thanks to CDM's generic nature, other studies are planned for inclusion in CDM's eCRF system, such as studies about optimal cytoreduction, pregnancies of unknown location, and fertility. CDM's modified user interface for conducting interrater agreement studies has been used for the six studies listed in Table 2.

**Table 2:** List of interrater agreement studies organized using CDM user interface modified for supporting such studies.

	Study	Phases	Reference
1	Improvement of interrater agreement through pictograms	With and without pictograms	[24], [15]
2	Endo-myometrial junction	1 and 2	[25-27], [16-18]
3	Polycystic ovaries	1, 2a, 2b	[28], [19]
4	Uterine anomalies	-	[29], [20]
5	IETA 2	-	Not yet published^a^
6	Contrast enhancement study	with and without enhanced contrast images	Not yet published^b^

^a^Data were collected between July 2012 and February 2013. Authors: L Valentin, A Installé, P Sladkevicius, D Timmerman, B Benacerraf, L Jokubkiene, A diLegge, A Votino, L Zannoni, and T Van den Bosch.

^b^Data were collected between May 2013 and February 2014. Authors: A Sayasneh, A Installé, D Timmerman, T Van den Bosch, T Bourne, S Guerriero, F Rizzello, LPG Francesco, MA Pascual, A Rossi, A Czekierdowski, A Testa, E Coccia, and A Smith.

### Data Analysis Component

CDM's data analysis APIs, and its Jython modules in particular, considerably simplify the derivation of machine-learning models from patient data. The integration of these capabilities into an eCRF system simplifies access to data, and the availability of the CRF definition simplifies preprocessing. Combined with the possibility to use these APIs interactively, CDM provides an excellent platform for rapid experimentation with different combinations of preprocessors and machine-learning algorithms in order to examine which combinations optimize predictive performance.

**Figure 6:**
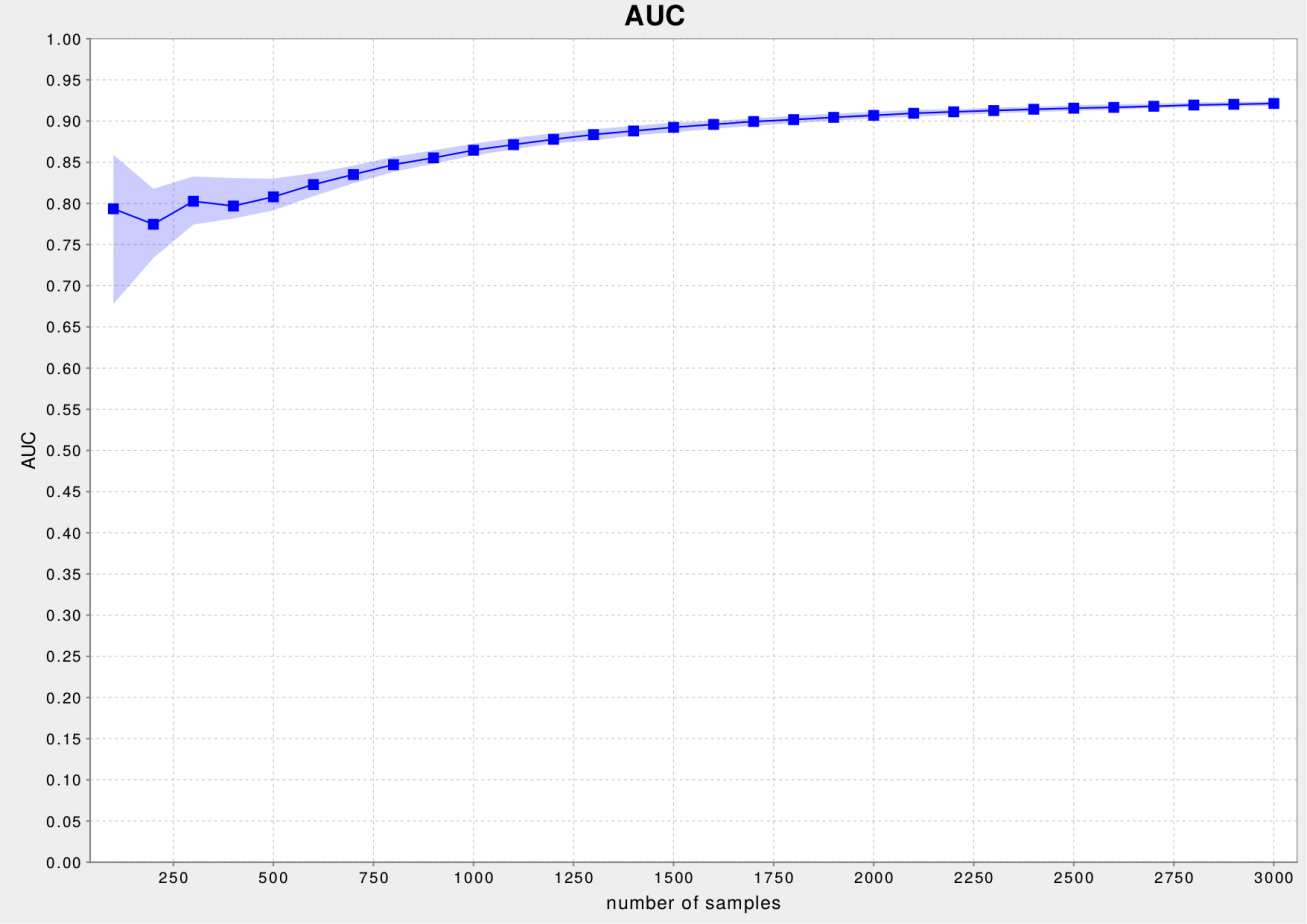
Learning curves, plotting predictive performance with respect to number of patient inclusions, can easily be generated using Clinical Data Miner (CDM)'s libraries. (Abbreviations: AUC=area under the ROC curve; ROC=receiver operating characteristic.).

CDM's APIs provide a method for easily generating learning curves; Figure 6 shows one of these. Such curves offer a clear insight into the evolution of a study's predictive performance as the number of patient inclusions grows, so that study coordinators can make an informed decision whether to continue or to terminate enrolling patients. As long as growing patient numbers result in marked performance improvements, patient data collection should continue in order to generate better models. By contrast, if the learning curves hit a plateau, or exhibit a slope that is negligible with respect to variability of performance results, patient recruitment should be terminated in order to avoid useless patient recruitment costs. The ability to optimize costs associated with patient enrollment results in more optimal patient numbers than Monte Carlo simulations [17] or rules of thumb [18] could provide, and has been very well received by the IETA consortium's steering committee.

**Table 3:** Breakdown per module of number of source lines of code (SLOC) and line and branch test coverage ratios, as determined by the sloccount and Cobertura programs, respectively.

	Production code	Test code	Line coverage	Branch coverage
	(SLOC^a^)	(SLOC)	n (%)	n (%)
cdm-common	5862	7023	1800/1957 (91.98)	459/486 (94.4)
cdm-server	15,260	28,109	5781/6250 (92.50)	1437/1577 (91.12)
cdm-client	3595	7607	1128/1269 (88.89)	133/146 (91.1)
cdm-client-gwt	4090	5123	957/1828 (52.35)	137/321 (42.7)
cdm-webapp	321	177	38/111 (34.2)	2/2 (100)
Total	29,128	48,039	-	-
Weighted average	-	-	9704/11,415 (85.01)	2168/2532 (85.62)

^a^Note that interfaces contribute to SLOC, but not to the number of lines analyzed for line coverage, leading to different counts for number of lines in the “Production code” and “Line coverage” columns.

### Software Development Methodology

Our TDD approach has delivered good test coverage, as is apparent from Table 3. Modules cdm-common, cdm-server, and cdm-client all have line and branch test coverage levels around 90%, guaranteeing high software quality. Modules cdm-client-gwt and cdm-webapp, responsible for binding graphical widgets to user interface logic, and therefore difficult to verify using unit tests, have lower test coverages. However, thanks to these latter modules' low complexity and infrequent changes, their lower test coverages do not negatively affect software quality.

### Survey

**Figure 7:**
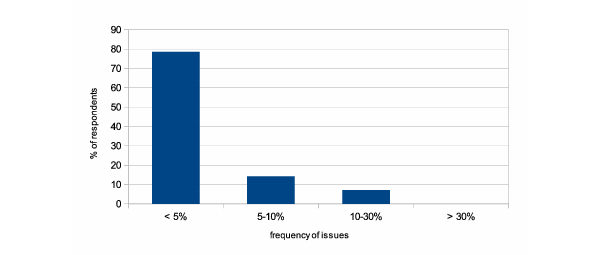
Distribution of respondents over different ranges of issue frequencies. A large majority, 79% (22/28), of survey participants experienced problems in less than 5% of their interactions with Clinical Data Miner.

Out of 42 clinicians contacted, 28 responded, resulting in a response rate of 67%. Survey results in Table 4 show CDM to be considered user friendly. Users particularly appreciate the possibility to integrate pictograms for clarifying questions. A large majority of users, 79% (22/28), experienced problems in less than 5% of interactions with CDM; Figure 7 shows this information. All respondents considered using CDM for the organization of their own studies.

**Table 4:** Average agreement levels with survey propositions among respondents.

Proposition	Average agreement^a^
CDM is user-friendly.	8.6
The layout of studies is clear.	8.6
The VAS^b^ is user-friendly.	8.1
CDM's VAS^b^ is a good alternative to a paper VAS^b^.	8.2
Pictograms help to clarify questions.	9.4
Pictograms help to differentiate multiple choice questions.	9.2
Pictograms next to multiple choice options will improve reliability.	9.3

^a^0 = no agreement; 10 = full agreement

^b^VAS = visual analog scale

## Discussion

### Principal Findings

We developed an eCRF software framework for supporting generic, multi-center clinical studies. Its high test coverage guarantees good software quality and good maintainability, while its modular architecture ensures the framework’s extensibility.

Its built-in data access, data preprocessing, and machine-learning capabilities streamline the clinical diagnostic model research workflow by eliminating data export and import steps, as well as by simplifying preprocessing. The possibility to access these capabilities through a Jython console provides an excellent platform for experimenting with different combinations of preprocessing and machine-learning algorithms.

The functionality to simplify the generation of learning curves enables study coordinators to assess whether to continue or to terminate data collection, providing better dataset size estimates than a priori application of rules of thumb or Monte Carlo simulations could deliver.

### Limitations

CDM does not currently support variable length array types, reducing its usefulness for longitudinal data capture. For bounded array sizes, presenting a fixed amount of fields representing the array can alleviate this issue.

CDM's data analysis capabilities are currently only accessible through a Java API or a Jython console, requiring programming expertise for their use.

Future work should solve these limitations, with better support for longitudinal data, and the integration of data analysis capabilities into CDM's user interface. The latter will, for example, enable study coordinators to visualize learning curves directly from within the user interface.

### Conclusions

The integration of data collection, preprocessing, and machine-learning in a single software framework simplifies the diagnostic model research workflow. The functionality for generating learning curves enables study coordinators to improve dataset size requirement estimates, also improving efficiency of clinical diagnostic model research.

## Abbreviations

API: application programming interface
CDM: Clinical Data Miner
COST: European Cooperation in Science and Technology
CRF: case report form
eCRF: electronic case report form
FOD: Federal Public Service
FWO: Research Foundation - Flanders
HIS: hospital information system
IETA: International Endometrial Tumor Analysis
IOF: Industrial Research Fund
IWT: Agency for Innovation by Science and Technology
SBO: Strategic Basic Research
SLOC: source lines of code
TBM: Applied Biomedical Research
TDD: test-driven development
VLK: Flemish League against Cancer
